# Development and performance test of an online blood sampling system for determination of the arterial input function in rats

**DOI:** 10.1186/s40658-014-0106-8

**Published:** 2015-01-14

**Authors:** Friedrich Roehrbacher, Jens P Bankstahl, Marion Bankstahl, Thomas Wanek, Johann Stanek, Michael Sauberer, Julia Muellauer, Thales Schroettner, Oliver Langer, Claudia Kuntner

**Affiliations:** Radiation Safety and Applications, Seibersdorf Laboratories GmbH, Seibersdorf, 2444 Austria; Department of Nuclear Medicine, Hannover Medical School, Hannover, 30625 Germany; Department of Pharmacology, Toxicology and Pharmacy, University of Veterinary Medicine, Hannover, 30559 Germany; Centre for Systems Neuroscience, Hannover, 30559 Germany; Biomedical Systems, Health & Environment Department, AIT Austrian Institute of Technology GmbH, Seibersdorf, 2444 Austria; Department of Clinical Pharmacology, Medical University of Vienna, Vienna, 1090 Austria

**Keywords:** Positron emission tomography, Scintillator, Photomultiplier, Coincidence detection, Arterial input function, Flow-through detector

## Abstract

**Background:**

For positron emission tomography (PET) kinetic modelling, an accurate determination of the arterial input function is required. In this study, a blood sampling system was developed and tested using different radiotracers in rats.

**Methods:**

The detector consists of pairs of lutetium yttrium oxyorthosilicate (LYSO) detectors, photomultiplier tubes and lead shield assembled within a steel casing working in coincidence mode. Rats were cannulated with microtubes in the femoral artery and vein for arterial blood sampling as well as administration of the PET tracers. Connected PTFE microtubes were centred between the LYSO crystals using a special holder. To enhance sensitivity, three layers with two coils were used. A flexible tube pump was used to ensure a constant blood flow. Performance of the detector was assessed with [^18^F]fludeoxyglucose (FDG), [^18^F]ciprofloxacin, (*R*)-[^11^C]verapamil, [^11^C]tariquidar, [^11^C]mephobarbital and [^11^C]MC113. Obtained input function curves were compared with manual samples drawn every 5 s during the first 3 min and further on at 5, 10, 20, 30, 40, 50 and 60 min after radiotracer injection. After manual sampling, an arterio/venous shunt was established. Shape and area-under-the-curve (AUC; Bq/μl*h) of the input functions were evaluated.

**Results:**

The developed detector system provided an absolute sensitivity of 6.5%. Maximum peak values agreed well between manual samples and the detector with a mean difference of −0.4% ± 7.0% (max 12.0%, min −9.9%). AUC values also exhibited an excellent correlation (*R* = 0.996) between manual sampling and detector measurements with a mean difference of 9.3% ± 9.7% (max 24.1%, min −3.2%). The system was able to measure peak blood activity concentration levels of 110 to 2,000 Bq/μl which corresponds to injected activities from 5.5 to 100 MBq depending on the used radiotracer, applied volume and weight of the animal.

**Conclusions:**

This study demonstrates that the developed blood sampling system can be used for *in vivo* small animal PET studies in rats in a reliable way. The usage of the systems enhances the accuracy of the input curve as handling of small blood samples especially with low activity (as for C-11) is prone to measurement errors. Additionally, the radiation dose of the experimenters can be reduced, as it is not required anymore to continuously draw samples where the personal is in close contact to the radioactive animals and blood.

## Background

Preclinical studies in laboratory animals have become an essential part in research in most areas of molecular biology, toxicology and drug discovery. Well-characterized rodent models have been developed for a range of diseases to offer the possibility of studying their fundamental mechanisms as well as to test potential drugs and therapies. Nowadays, tracer kinetic modelling is often used in positron emission tomography (PET) studies to quantify receptor density, receptor occupancy or metabolic activity or to assess the pharmacokinetics of new radiolabelled molecules [1]. For calculation of the tissue response function determination of an accurate arterial input function is required. In small animal studies, arterial sampling is limited by small blood volume of rodents, and the irreversible blood loss bears an extra burden for the animal and may perturb the physiology and thus confound the experimental outcome [2,3].

Several methods to measure an input function have been proposed and applied in human and rodent studies. These include the measurement of an image-based input function [4,5], the use of a standardized input function based on population curves [6], the use of transcutaneous measurements [7] and the use of an intra-blood-vessel probe for direct positron detection [8,9]. Although all these approaches have their advantages, reliability of the input function when using new tracers or diseased animal models must be validated by comparison with arterial blood sampling data.

Several investigators have proposed flow-through blood sampling detectors. Advantages of continuously measuring blood radioactivity are much shorter sampling times, higher statistical accuracy and reduced manual labour, and moreover reduction of radiation dose to the technologist drawing blood samples. A blood sampling flow-through detector can rely on detection of emitted positrons or single and/or coincidence detection of annihilation photons from blood in the catheter lumen. The advantage of photon detection is that sensitivity does neither depend on positron energy nor on tubing and detector casing so that even a low positron energy emitter such as ^18^F can be measured. Hutchins et al. [10] and Kanno et al. [11] used flow-through plastic scintillators to detect positrons. Both found the necessity to shield the detector from high levels of background radiation from the patient. Other authors have described systems based on detection of one or both annihilation photons, at cost of a higher sensitivity to background radioactivity. Nelson et al. [12] reported on a sodium iodide (NaI)-based detector whereas Ranicar et al. [13] and Eriksson et al. [14,15] reported on bismuth germanate (BGO)-based detectors applicable to human PET studies. In the later publication, a well-shaped scintillator geometry was used that allowed counting both 511- and 1,022-keV summation peaks. A pico-count flow-through detector where no dispersion correction is needed was described by Votaw and Shulman [16] and is operating in coincidence detection mode of the two 511-keV gamma-ray photons with BGO detectors. At about the same time, a BGO/photomultiplier tube (PMT) system consisting of four detector units assembled in a whirlpool shape for rabbit PET studies was proposed by Tadokoro et al. [17]. Because of the large field of view (FOV) of 20 mm Ø × 30 mm, a half-looped extension tube was used for blood sampling. One of the first applications of a flow-through system in preclinical studies was demonstrated by Ingvar et al. [18]. Here, an automatic blood sampling system developed for human studies [14] was used for a rat study operating via a femoral arterio-venous shunt. Recently, a phoswich detector based on the gadolinium oxyorthosilicate (GSO) scintillator with different decay times has been proposed by Yamamoto et al. [19], where the front layer detects positrons and background gamma-ray photons, whereas the back layer detects background gamma-ray photons only.

In the present study, we developed and evaluated an online blood sampling detector based on the lutetium yttrium oxyorthosilicate (LYSO) scintillator. This scintillator was chosen due to its high stopping power, high light yield and fast decay time. The detector was evaluated in rat animal PET studies and relies on counting radioactivity in arterial whole blood flowing through an arterio-venous shunt leading from the femoral artery to the ipsilateral femoral vein. By coincident counting, background rejection works well and the detector can be placed very close to the small animal PET scanner with only minimal shielding. To assess response for different nuclides (^18^F and ^11^C), six different radiotracers: [^18^F]fludeoxyglucose (FDG), [^18^F]ciprofloxacin, (*R*)-[^11^C]verapamil, [^11^C]tariquidar, [^11^C]mephobarbital and [^11^C]MC113 were measured and compared with manually sampled blood curves.

## Methods

### Flow-through detector design

#### System description

The proposed online blood sampling detector consists of a detector unit, a peristaltic pump and a main unit controlled by dedicated software. The whole system is mounted on a trolley which can be moved as close as possible to the small animal PET scanner. The detector unit includes the coincidence detector and shielding. The main unit includes a PC and all electronics (NIM-bin) required for signal threshold adjustment, amplification and coincidence detection. The peristaltic pump (Ismatec Ecoline VC-MS/CA4-12, ISMATEC, Glattbrugg, Switzerland) ensures constant blood flow and can accommodate speed performance from 10 to 5,400 μl/min using a pump tube with 0.5 mm inner diameter. All measurements described in this article were carried out using a sampling time of 1 s and a withdrawal speed of the pump of 840 μl/min, which corresponds to the arterial blood flow of an anesthetized cannulated rat assessed in independent measurements (data not shown). The software was customly designed to set experiment parameters, process recorded data and display the blood time-activity curve in real time. It includes the required data correction described in the ‘Software’ section.

#### Detector geometry

**Figure 1:**
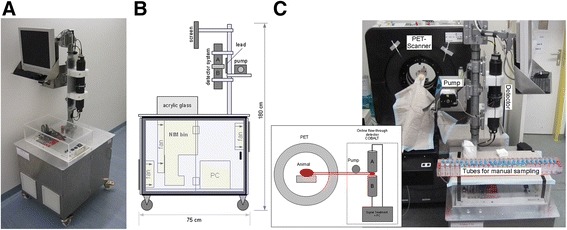
**Picture** **(A)** **and sketch** **(B)** **of the complete measurement device.** In **(C)**, the flow-through blood detector is shown in operation mode next to the PET scanner and connected to the venous and arterial catheters of a rat.

**Figure 2:**
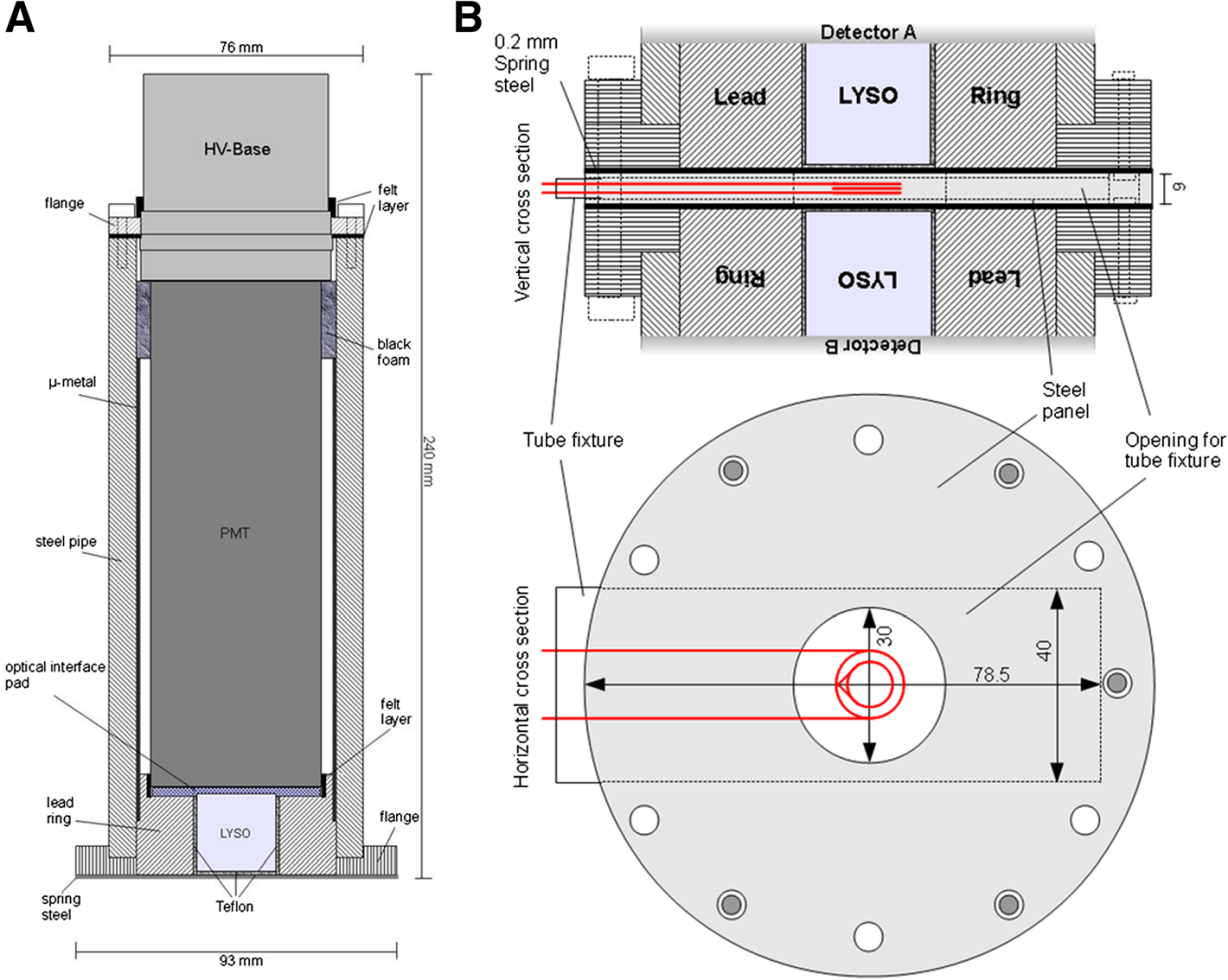
**Schematic drawings of the developed flow**-**through blood detector.** In **(A)**, a single detector is outlined whereas in **(B)**, the inner part for the coincidence detection and the opening for the tube fixture are shown.

The online blood sampling detector is based on coincidence gamma-ray photon detection using two opposed LYSO scintillators (Lu_1.8_Y_0.2_SiO_5_:Ce, Hilger Crystals, Unit R1 Westwood Estate, Margate, Kent, CT9 4JL, England) each coupled to a photomultiplier tube (PMT; XP20D0, Photonis, Brive, France). Differences in their gain caused a shift in pulse height spectra and were corrected by using individually adjusted high voltage supplies to the two PMTs (1,165 and 985 V). A picture and schematic drawing of the developed detector are shown in Figure 1. The cylindrical crystal has a size of 2.54 cm in diameter and a length of 2.54 cm. The crystal is covered at all sides (except of the coupling side to the PMT) with a thin reflection foil (Radiant Mirror VM-2000, 3M, St. Paul, MN, USA) and surrounded by two layers of Teflon (each 500 μm) to improve reflection and collection of scintillation photons. The covered scintillator is positioned inside a ring of lead (17 mm) which is used as shielding against background radiation and to ensure reproducible position. On the entrance window of the PMT, an optical interface pad (EJ-560, Eljen Technology, Sweetwater Texas, USA) is attached in order to level out imperfections in the construction of steel pipe and flange (see Figure 2). To further improve optical contact, optical grease is used between the interface pad and the scintillator. Since the crystal is slightly taller than the surrounding lead contact pressure (brought onto the PMT by the outer metal coating) between entrance window and crystal squeezes the pad, which (together with grease) leads to an optimal optical coupling. The PMT is surrounded with a μ-metal cylinder and a layer of felt, which functions as light sealing from outside photons, and is used as an additional isolation layer since the photocathode is at high voltage. The whole detector is finally packed in a varnished steel pipe to ensure mechanical stability. It furthermore serves as an additional shielding from outside background radiation (e.g. the animal itself). For further shielding of radiation from the animal, an additional 1-cm lead shield is mounted next to the detector system.

For coincidence detection, two detectors are placed in opposition with a small slot opening of 7 mm for the positioning of the catheter tube in between. Withdrawn blood is pumped through tubing, which is run between two detectors in a geometry designed to optimize coincidence counting rate for 511-keV photons.

#### Measurement geometry

**Figure 3:**
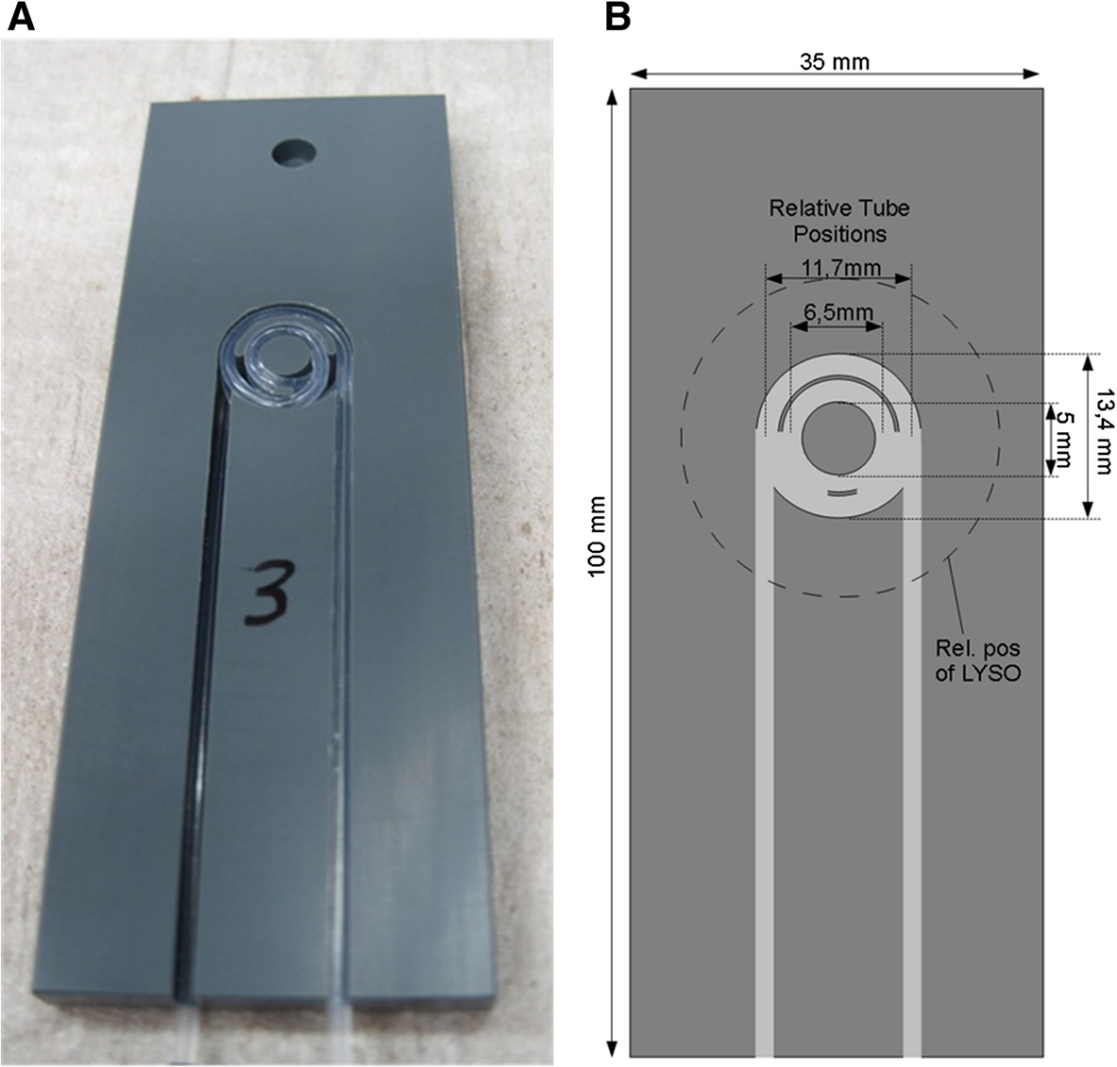
**Picture** **(A)** **and detailed sketch** **(B)** **of the tube fixture.** The Teflon tube is spooled in two coils (inner and outer coil) each consisting of three layers and kept in this fixed and reproducible position by press-fitting into the milled channels. The sketch also displays the position of the LYSO crystals (dashed circle) when the tube fixture is inserted completely into the opening of the detector system.

The blood sample is carried between two detectors inside plastic tubing which is held in a reproducible fixed position by press-fitting into a milled channel in a PVC fixture (see Figure 3). The tubing is spooled in two coils each consisting of three layers resulting in a blood volume of 33.4 μl inside the detection area. The outer shape fits exactly into the insert slot of the steel panel between the two detectors and runs up to a stop to ensure consistent counting geometry between separate experiments. Hence, neither the tube fixture nor the tube itself can move during a measurement. Since furthermore the tube can only be placed in one way in the tube fixture, the geometry is stable and reproducible for each measurement. As the blood sample is completely contained in the Teflon tube, there is little possibility of any biological contamination.

#### Signal processing

**Figure 4:**
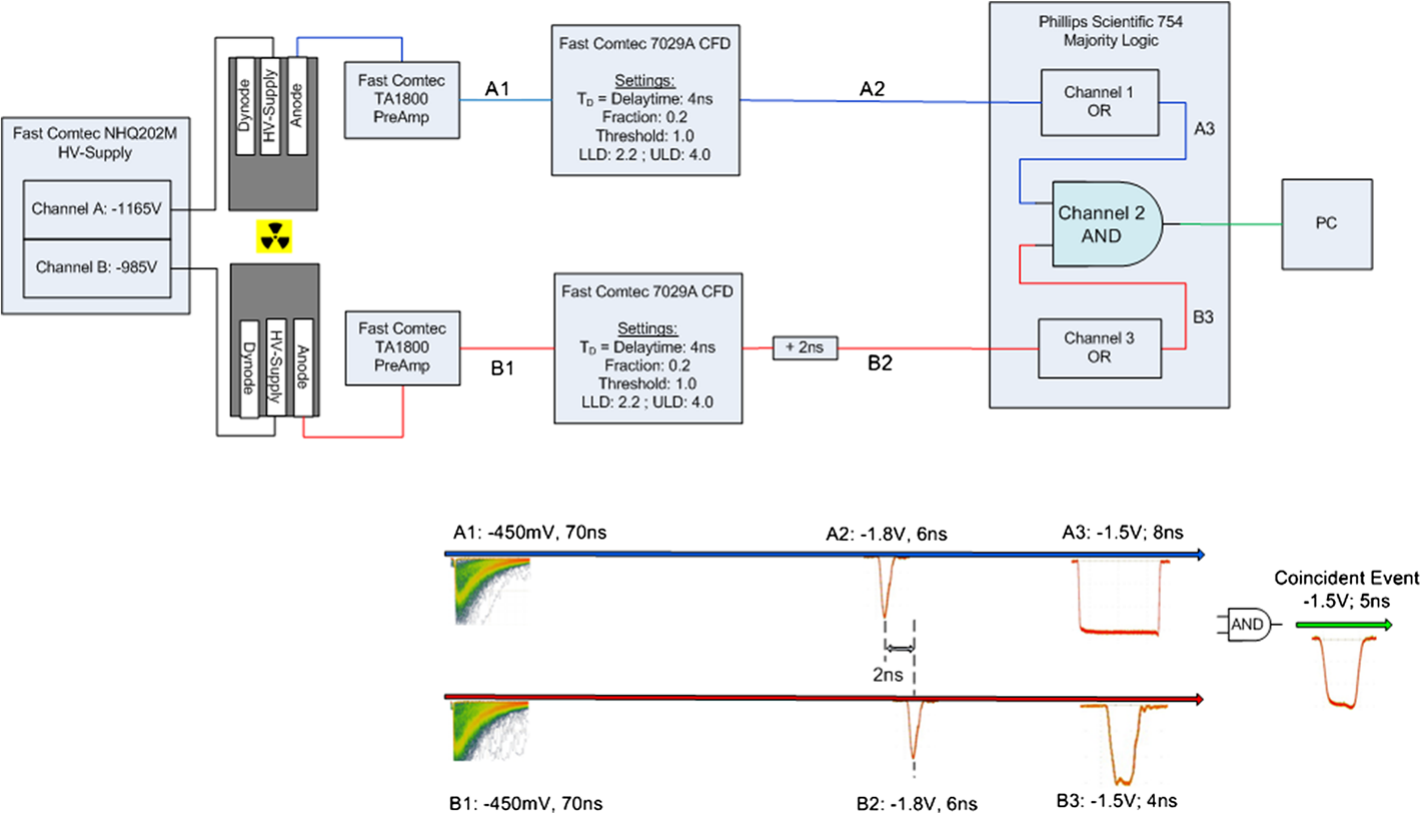
**Block diagram of the signal processing.** The analogue output pulse of the PMT is first amplified and then converted by the CFD into a digital signal. The signal of channel B is delayed by 2 ns. Within the majority logic, signal A is stretched to 8 ns while signal B is stretched to 4 ns. If both signals occur at the same time, channel 3 of the majority logic (configured as logic AND) produces a digital output pulse. This pulse is finally regarded as annihilation event.

The block diagram of the signal processing is illustrated in Figure 4. Amplified PMT outputs (Fast Pulse Timing Preamplifier TA1800, FAST ComTec, Oberhaching, Germany) are fed directly into constant fraction differential discriminators (7029A, FAST ComTec, Oberhaching, Germany) where the signals are filtered for their peak voltages. Only those with a peak voltage between the lower level discriminator (LLD) at −360 mV and the upper level discriminator (ULD) at −630 mV are used to trigger a digital output pulse. These voltages correspond to energies of 415 and 680 keV. Both pulses are then sent to the coincidence unit (Quad Majority Logic 754, Phillips Scientific, Mahwah, NJ, USA); however, compared to channel A, the pulse of channel B is delayed by 2 ns. Within the logic unit, the rising edges of the input pulses are used to trigger an 8-ns pulse in channel A and a 4-ns pulse in channel B. The coincident time window is therefore 12 ns. If pulse B occurs within the duration of pulse A, the logic AND produces a 5-ns output pulse representing the coincident pulse. These fast timing constraints ensure that accidental coincidences are kept to a minimum so that the detector can measure valid events, even in the presence of substantial background radiation from the animal. The coincident pulse is finally transmitted to the PCI card in the PC where the counter is increased.

#### Software

Each measurement is controlled by software especially written for this device. The software, written in Visual Basic, leads the user through a standard setup sequence, during which various study descriptions (e.g. isotope used) are entered and a sampling time sequence is set up. Besides its control and display features, it offers a variable integration time (VIT) smoothing the time-activity curve (TAC) as the count rate gets low. The VIT function calculates the relative uncertainty of *N* consecutive measurements based on their statistical fluctuation. The calculation is carried out by fitting a linear function to the log values after the peak (linear regression). The result is then compared to the coefficient of variation for each measurement defined as the reciprocal square root of its mean. If the outcome is smaller than the coefficient of variation, the software displays the last calculated value. As the statistical fluctuations increase with lower count rate, the number of consecutive measurement values is increased by steps of 2 up starting from *N* = 4 to a maximum value of *N* = 20. Hence, blank value and detection capability depend on the integration time chosen by the program during a measurement and the isotope used for investigation. The latter reason originates from the different positron emission probabilities. The software includes half-life correction, blank value correction and accounts for the time delay of the radioactive blood from the animal to the detector. Simultaneously with data acquisition, the arterial time-activity curve is visualized on the computer screen as well as saved to a csv file. Although the counts recorded can be used directly to derive the TAC, these features of the software make the whole system much more practicable and efficient.

#### Energy resolution and count rate capability

The energy resolution of the system was measured with a ^68^Ge point source placed in the centre between the LYSO detectors. The measured signal must be corrected for efficiency of the detector, physical decay during transit time and dispersion of radioactivity during the transit. The system was calibrated for defined energy settings of 415 and 680 keV for lower- and upper-level threshold with a low concentration of [^18^F]FDG in an aqueous solution with an activity concentration of 2.1 MBq/ml. The high count rate capability was determined by using [^18^F]FDG with an initial concentration of around 75 GBq/ml. The measurement was followed for 11 half-lives. At lower count rates (towards the end of the measurement), the system can process all pulses. However, the larger the count rate is (at the beginning of the measurement), the more pulses are lost. By extrapolating low count rate data to high count rate data, assuming a linear system, an ideal measurement curve can be calculated. The ratio between real and ideal curve gives the lifetime fraction.

#### Minimum detectable activity (MDA)

When calculating the detection limit, the net counts *N*_*S*_ (counts caused by the source only without background counts) are compared to a threshold to determine if a source is present or not, typically with 95% confidence. The critical limit *L*_*C*_ is defined so that, if *N*_*S*_ < *L*_*C*_, we may conclude that no source is present with a false-positive rate no larger than 5%. The detection limit *L*_*D*_ is defined so that, if *N*_*S*_ > *L*_*D*_, we may conclude that a source is present with a false-negative rate no larger than 5% [20].

The MDA is defined as the minimum amount of radioactive material necessary to yield the detection limit *L*_*D*_ and can be calculated as:

$$ \mathrm{M}\mathrm{D}\mathrm{A}=\frac{N_T-{N}_B}{f\epsilon VT} $$

where *N*_*T*_ and *N*_*B*_ are the mean values of the total counts (caused by a source including background) and by background only, *f* is the emission probability of positrons for the corresponding isotope, *ε* the sensitivity, *V* the measurement volume and *T* the integration time. Consequently, as the integration time increases, the MDA gets lower. This formula was adapted from the equation given by Knoll [21] to include the measurement volume.

### *In vitro* evaluation of the flow-through detector

Before *in vivo* evaluation, different tubing materials were tested, as the system should be able to work with different kinds of tracers. Central nervous system (CNS) tracers are usually very lipophilic and show a high adsorption to different materials like the inner walls from tubes. We tested three different tubing materials (all obtained from LIQUID-scan, Ueberlingen, Germany): 1) Tygon standard tubing (R3607, ID = 0.51 mm, OD = 2.33 mm), 2) Tygon silicon tubing (Pt3350, ID = 0.51 mm, OD =2.35 mm) and 3) a combination of Teflon®PFA (ID = 0.5 mm, OD = 1.55 mm) and Pharmed®BPT (ID = 0.51 mm, OD = 2.21 mm) tubing, as the Teflon®PFA tubing does not work inside the peristaltic pump. A shielded vial was filled with tracer ((*R*)-[^11^C]verapamil or [^18^F]FDG) mixed with NaCl or heparinized rat blood. The detector was started, and the tube was positioned into the vial. To study adsorption of the radiotracer to the inner walls, the tube was removed from the vial after a certain time and all liquid was emptied into the waste vial. Some minutes later when the tubing was free of liquid, the tube was positioned in a vial containing NaCl solution. Curves were decay-corrected to the beginning of acquisition.

With the final setup, dispersion measurements for ^18^F and ^11^C studies were performed according to the method described by Munk et al. [22]. Large blood samples were drawn from rats and portioned into 10-ml vials. For the ^18^F study, 12.9 and 34.3 MBq of [^18^F]FDG was incorporated into two blood-filled vials. For the ^11^C study, 47.8 and 23.6 MBq of (*R*)-[^11^C]verapamil was mixed into two blood-filled vials. Two vials each (with and without tracer) were used to generate square step functions for each nuclide. The square function was generated by positioning the tube from the flow-through detector into the tracer-free vial and starting sampling for 60 s; then quickly, the tube was placed into the tracer vial and events were recorded for 90 s. Afterwards, the tube was placed again into the tracer-free vial, and sampling was continued for around 50 s. The obtained curves were decay-corrected to the beginning of acquisition. The dispersion for the increasing part was modelled as a monoexponential function [22].

$$ D(t)={\left\{{}_{A\left(1-{e}^{-k\left(t-T\right)}\right)}^{0,}\right.}_{\kern0.95em T\ \le\ t\ \le\ T+\varDelta T}^{t<T} $$

where *A* is a constant activity concentration, *k* (s^−1^) is the dispersion rate constant, *T* is the transit time defined at the time point where the tube was positioned into the tracer vial and *ΔT* is the sampling duration from the tracer vial. The dispersion time constant was then calculated as *τ* = 1n(2)/*k* [16,23].

### *In vivo* evaluation of the flow-through detector

#### Animals

Adult female Sprague-Dawley rats (Harlan Nederland, Horst, Netherlands) weighing 270 to 350 g were used for this study. Animals were kept under controlled environmental conditions (22°C ± 1°C; 40% to 70% humidity; 12-h light/dark cycle) with free access to standard laboratory animal diet and tap water. Before being used in the experiments, the rats were allowed to adapt to the new conditions for ≥1 week. The study was approved by the local Animal Welfare Committee, and all study procedures were performed in accordance with the Austrian Animal Experiments Act. All efforts were made to minimize pain or discomfort as well as the number of animals.

#### Chemicals and radiotracers

Unless otherwise stated, all chemicals were of analytical grade and obtained from Sigma-Aldrich Chemie GmbH (Steinheim, Germany) or Merck (Darmstadt, Germany) and used without further purification. Isoflurane was obtained from Baxter Vertriebs GmbH (Vienna, Austria). Synthesis and quality control of [^18^F]FDG was performed by using standard methods [24]. Synthesis of (*R*)-[^11^C]verapamil, [^11^C]tariquidar, [^18^F]ciprofloxacin, [^11^C]mephobarbital and [^11^C]MC113 was performed as described before [25-29].

#### Experimental procedure

Prior to each experiment, the animals were placed in a chamber containing 2% isoflurane in oxygen. When unconscious, animals were taken from the chamber and kept under anaesthesia with 1.8% to 0.6% isoflurane administered via a mask during the whole experiment. During the experimental procedure, the isoflurane level was adjusted depending on depth of anaesthesia. Animal monitoring was performed visually by assessment of breathing rate and perfusion of acra, as well as by testing reflexes. A humidifier was used to moisten the gas mixture before supplying it to the animal, thus preventing impairment of air passages. Femoral artery and femoral vein were cannulated with a medical micro-volume tubing made of Tygon (S-54-HL Kleinfeld Labortechnik, Gehrden, Germany, ID = 0.508 mm, OD = 1.524 mm). During surgery, animals were i.v. administered with 0.9% (w/v) aqueous saline solution containing 20 IE/ml sodium heparin to prevent clotting of catheters. Body temperature was monitored and kept close to 37°C. Four out of 11 studied animals underwent a dynamic 60-min small animal PET scan using a microPET Focus 220 scanner (Siemens Medical Solutions, Knoxville, TN, USA). Vital parameters (temperature and respiration rate) during the scan were monitored using the SAII 1025L monitoring and gating system (Small Animal Instruments, Inc, Stony Brook, NY, USA).

The arterial catheter having a length of approximately 40 cm was connected with the tubing from the flow-through detector, which was flushed with 0.9% (w/v) aqueous saline solution containing 20 IE/ml sodium heparin. The pump was started, and saline was collected in a vial. After the whole system was filled with blood, the tubing from the detector was connected with the venous catheter creating an extracorporeal arterio-venous shunt. The total extracorporeal blood volume in the tubing systems (catheters plus tubing from the detector) was around 250 μl. Shortly before radiotracer injection, the shunt was disconnected on the venous side and the radiotracer was administered as an i.v. bolus via the femoral vein over 46 ± 18 s. The injected radiotracers, radioactivity and volumes are summarized in Table 1.

**Table 1:** **Overview of the injected radiotracers and number of animals used**

**Tracer**	**Number of animals**	**Injected activity** **[MBq]**	**Injected volume** **[μl]**
[^18^F]FDG	1	6.9	300
[^18^F]ciprofloxacin	3	5.5	950
		11.6	1,000
		17.3	700
(*R*)-[^11^C]verapamil	2	32.1	250
		57.9	300
[^11^C]tariquidar	2	39.7	300
		41.6	250
[^11^C]mephobarbital	2	55.1	250
		57.6	250
[^11^C]MC113	1	97.4	250

Repeated arterial blood sampling was performed on the arterial side after the blood had passed the detector. During the first 3 min after radiotracer injection, around 20-μl arterial blood samples were continuously collected every 2 to 3 s using pre-weighted sample tubes. Afterwards, the shunt was closed. Further manual 40-μl blood samples were taken at 5, 10, 20, 30, 40, 50 and 60 min after injection. Radioactivity in blood samples was measured in a 1-detector Wallac gamma counter (Perkin Elmer Instruments, Wellesley, MA, USA), which was cross-calibrated with the PET camera.

Blood radioactivity data were corrected for radioactive decay and expressed as Bq/μl. The area under the blood time-activity curve (AUC; in Bq/μl*h) was calculated using the OriginPro 8.5.1G software package (OriginLab Corporation, 350 Northampton, MA, USA).

## Results

### Physical characterization

#### Detector performance and detection limits (MDA)

The energy resolution was 23% (full width at half maximum (FWHM)) at 511 keV. The rise and decay time of pulses were approximately 6 and 60 ns, respectively.

From purely geometrical arguments, it can be shown that the two crystals subtend about 74% of the azimuthal angle around the point source at a distance of 11 mm between both crystals corresponding to a solid angle fraction of 60.2%. The probability for a 511-keV photon to be absorbed in the 2.54-cm LYSO crystal is 86% (as given by the manufacturer). The trigger efficiency is based on the trigger threshold used in the readout electronics (415 keV) and the estimate of inter-crystal scatter and edge effects. With a LLD of 415 keV and an ULD of 680 keV, a sensitivity of 6.5% for the measurement geometry described in the ‘Measurement geometry’ section was measured.

**Figure 5:**
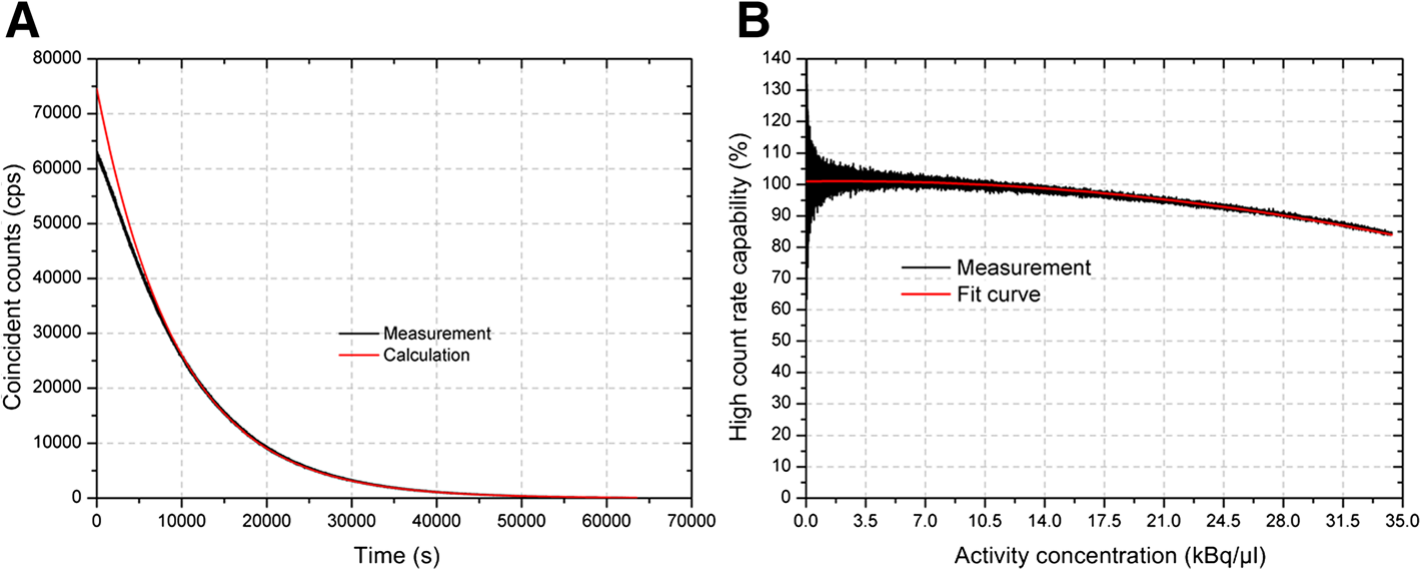
**Evaluation of the systems dead time behavior.** **(A)** Comparison between the measured and the ideal coincident count rate curve. The ratio between the real and the ideal calculated curve gives the lifetime fraction illustrated in **(B)**.

The dead time behaviour of the detection system was evaluated by comparing the measure coincident counts to the ideal count rate curve. Figure 5A displays the measured data compared to the calculated data while in Figure 5B the relation between these data (i.e. the lifetime fraction) as a function of the activity concentration is plotted. Figure 5B shows that above an activity concentration of about 8 kBq/μl, the system cannot process all counts and, consequently, the data need to be corrected for the dead time.

The detection capability is limited by the self-activity of LYSO crystals and background activity from the animals. Natural lutetium in a LYSO (Lu_1.8_Y_0.2_SiO_5_:Ce) crystal contains 2.6% of its radioactive isotope ^176^Lu [30] causing an activity of approximately 3,500 Bq for a 2.54 × 2.54 cm cylindrical crystal. ^176^Lu decays via beta emission to the 597 keV excited state of ^176^Hf which relaxes to its ground state by emitting a cascade of three gamma-rays with an energy of 307, 202 and 88 keV [31]. With a LLD of 415 keV and an ULD of 680 keV, a natural background of about 1,400 counts per second for each detector was measured. However, as the coincidence window was set to 12 ns, the probability for detecting two decays (each in one detector) within this time range is almost zero. Consequently, the coincidence count rate caused by self-activity of 1.3 ± 1.1 cps using the PVC tube fix is caused almost exclusively by detecting one single decay (the beta particle is detected in the detector where the decay occurs while either all three or the two highest energy gamma-rays are detected in the other one).

**Figure 6:**
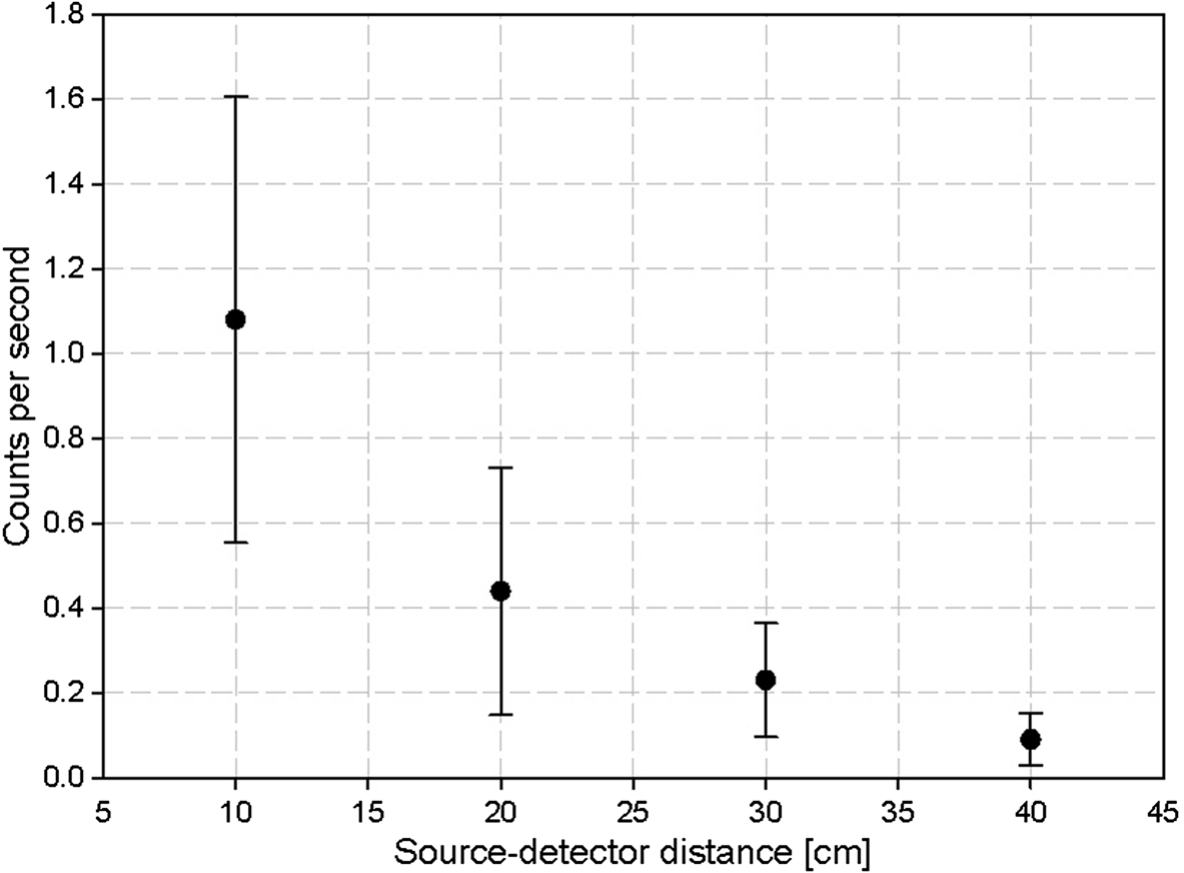
**Measurement of the influence of background activity in relation to the distance from the detector.** A 185-MBq perturbation source was used for this measurement, and the coincidences were recorded.

The other source of background activity is the animal itself. Maximum activity administered to the animal is usually smaller than 185 MBq (5 mCi) as the peak NEC value is 170 MBq for the Focus 220 (250 to 750 keV and 6-ns timing). Figure 6 shows the number of error counts depending on the distance of a 185-MBq perturbation source if the flow-through detector was placed such that the additional lead shield (1 cm) is right between animal and detector. As the common setup requires a distance of about 40 cm between animal and detector the perturbation-error count rate is 0.1 ± 0.05 cps. Taken together, the total blank value can be calculated 1.4 ± 1.1 cps.

The MDA varies between 1.0 and 3.6 Bq/μl for ^11^C and between 1.0 and 3.7 Bq/μl for ^18^F depending on the used integration time. The lower values correspond to the maximum integration time of 20 s while the higher values correspond to an integration time of 1 s.

Due to its excellent timing properties, the device can process all events up to an activity concentration of 8 kBq/μl. Above this level, the lifetime fraction decreases to e.g. 90% for an activity concentration of 28 kBq/μl.

#### Temperature dependence

As LSO and therefore also LYSO are known to depend on temperature [32], influence on the light yield was studied for the given setup. The light yield decreases with increasing temperature and therefore electric load and peak voltage of the output signal becomes lower, leading to decreased signals above LLD level. Thus, count rate decreases as temperature rises. Various measurements by the authors have shown that the actual correlation (including the temperature dependence of the PMT) is given by *∆*CR = −0.5% / *∆T*, where *∆*CR is the relative change of count rate and *∆T* is the change of temperature. As the instrument is operated in an air-conditioned laboratory, the influence of the temperature on the count rate can be neglected.

#### Time resolution

The time resolution (FWHM) of 550 ps of this system (including constant fraction discriminators (CFDs)) was determined using the TDC setup of the Multiparameter Data Acquisition System MPA-3 together with a Dual Timing ADC 7072T (both by FAST ComTec, Oberhaching, Germany). Two reasons are mainly responsible for that: variations of the PMT transit time and different rise times of the analogue input signal at the CFD. However, these different rise times can be compensated partly by using the constant fraction technology. For these PMTs analogue output signals, a fraction of 0.2 and an external delay of 4 ns were chosen as ideal settings at the CFDs to optimise the timing resolution.

### *In vitro* evaluation

**Figure 7:**
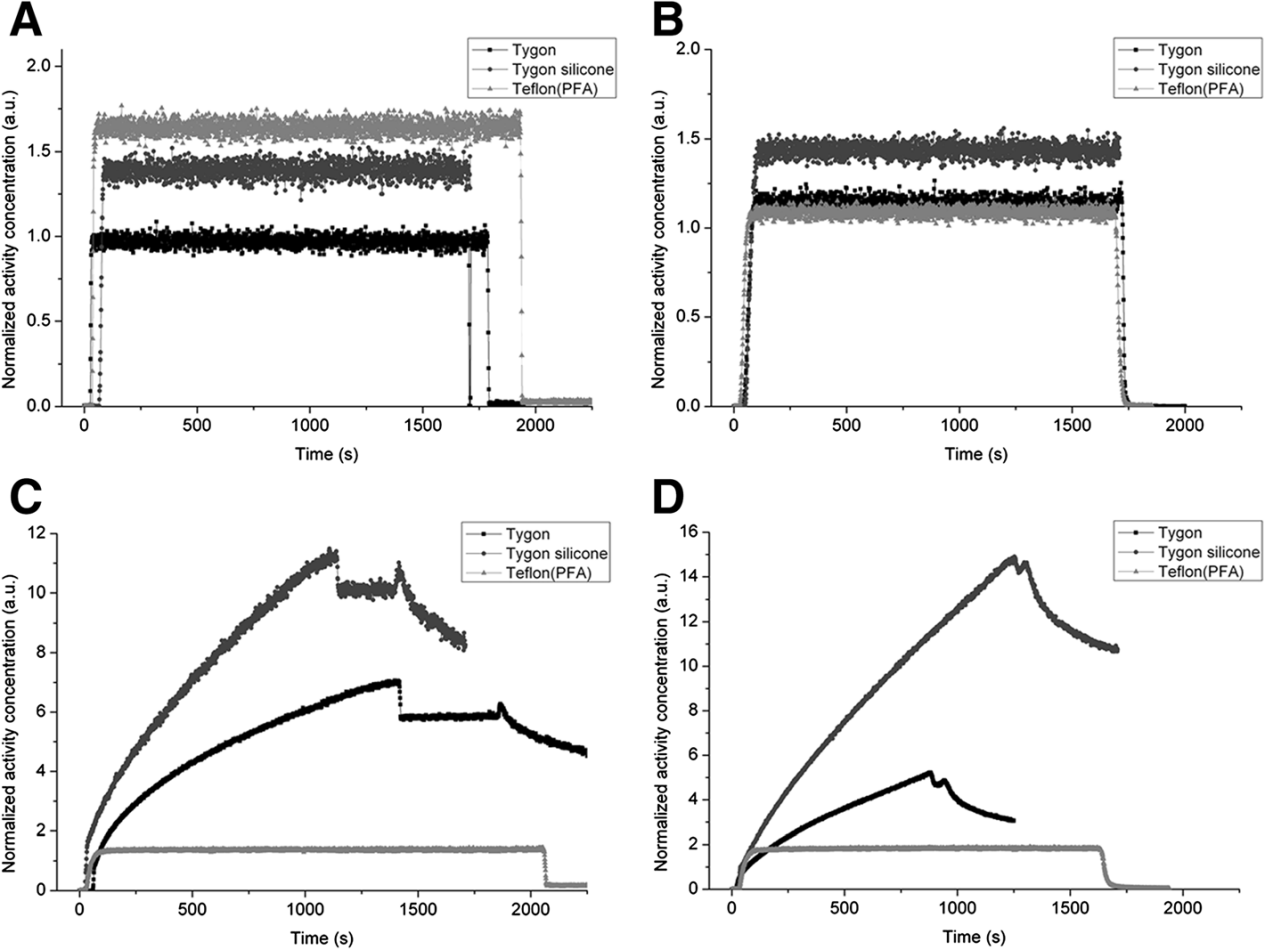
***In vitro*** **results when filling the flow**-**through detector with a known radioactivity concentration varying between 450 and 3,500 Bq/μ** **l. (A)** [^18^F]FDG in NaCl, **(B)** [^18^F]FDG in blood, **(C)** (*R*)-[^11^C]verapamil in NaCl and **(D)** (*R*)-[^11^C]verapamil in blood. The flat parts in (C) and (D) occurred when the tubing was emptied from all liquid. Afterwards, it was flushed with NaCl (decreasing part of (C) and (D). All TACs are corrected for radioactive decay to the start of acquisition.

Results from *in vitro* evaluation of (*R*)-[^11^C]verapamil and [^18^F]FDG mixed in NaCl or blood can be seen in Figure 7. Significant (*R*)-[^11^C]verapamil adhesion to the tubing walls of Tygon standard tubing (R3607) and Tygon silicon tubing (Pt3350) was observed both with (*R*)-[^11^C]verapamil mixed in NaCl and also in blood. Adhesion of (*R*)-[^11^C]verapamil to the inner walls of the Tygon silicon tubing was even stronger as compared to the Tygon tubing alone. The adhesion of this lipophilic tracer to the inner walls led to a constant increase in the count rate over time. When replacing the radioactive solution with air, a constant count level was observed in the detector (compare flat part of Figure 7C,D) pointing to radioactivity sticking to the inner walls. Even when flushing the system with NaCl solution (decreasing part of figure 7C and 7D), the count rate only slowly decreased to a lower level. No adhesion was found for [^18^F]FDG for the Tygon silicon and Tygon tubing. Here, for all tested tubing materials, the count rate quickly returned back to the background level, when the system was emptied. For the Teflon tubing (PFA), no adhesion was observed for [^18^F]FDG as well as for (*R*)-[^11^C]verapamil.

**Figure 8:**
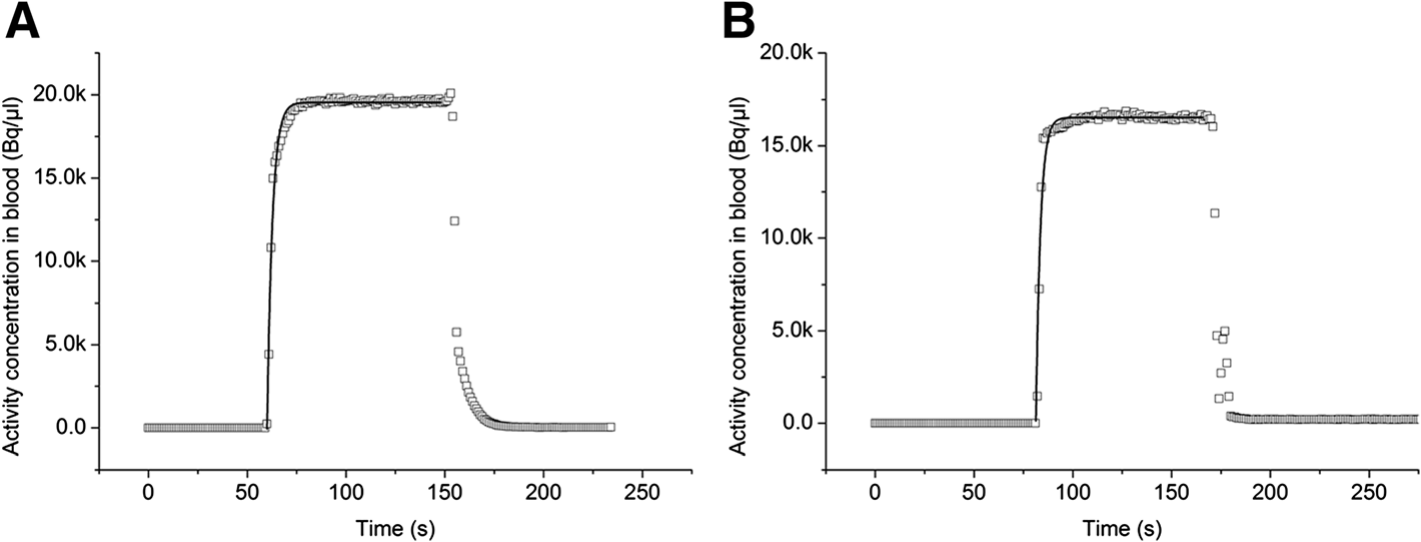
**Example of square functions measured by the online blood sampling system using** **(A) [** ^**18**^ **F]** **FDG and** **(B) (** ***R*** **)-[** ^**11**^ **C]** **verapamil.** The obtained dispersion curves were fitted using a monoexponential function (white squares are measurements and the line corresponds to the best fit). Measurements were performed using 34.3 MBq [^18^F]FDG mixed in 1.59-ml blood and 47.8 MBq (*R*)-[^11^C]verapamil mixed in 2-ml blood. Both dispersion curves were corrected for radioactive decay to the start of acquisition.

The dispersion in the online sampling system was measured using a step function created with [^18^F]FDG and (*R*)-[^11^C]verapamil. For all the dispersion measurements, the Teflon tubing setup was used. The measured response to the step function is shown in Figure 8. For both used tracers, we obtained a dispersion effect for the two measurements. With a measurement volume of 33.4 μl and a flow rate of 840 μl/min, it takes around 2.4 s to fill the detection volume. From the two [^18^F]FDG measurements, monoexponential fits yielded dispersion rate constants of 0.325 and 0.372 s^−1^. This corresponds to dispersion times of 2.1 and 1.9 s. For the (*R*)-[^11^C]verapamil measurements, dispersion rate constants of 0.427 and 0.416 s^−1^ were obtained from the fits corresponding to dispersion times of 1.6 and 1.7 s, respectively.

### *In vivo* evaluation

**Figure 9:**
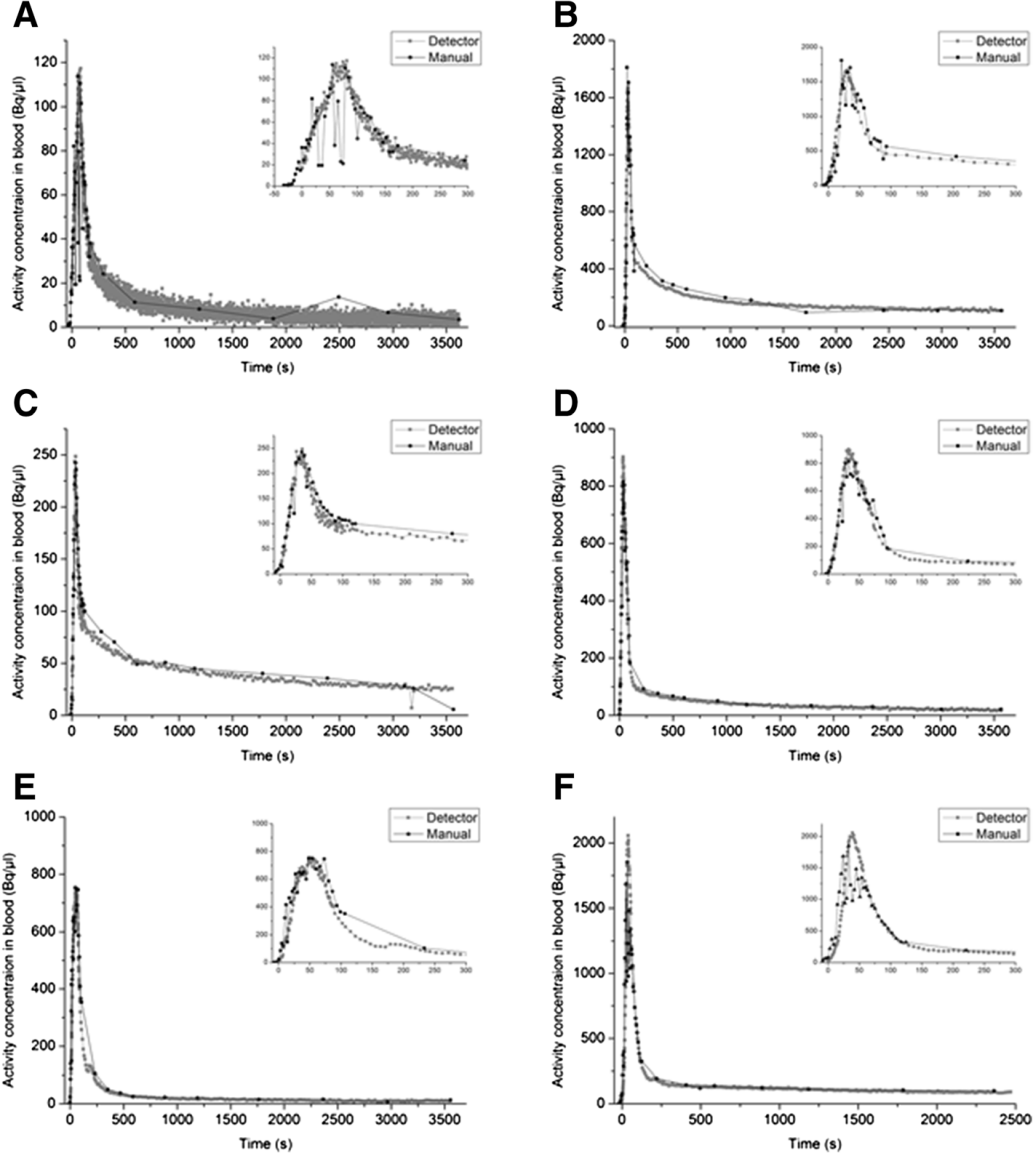
***In vivo*** **evaluation of the flow-through blood detector.** Comparison between the blood TAC obtained with the developed flow-through detector (grey squares) or obtained by manual sampling (black circle) after injection of **(A)** 5.5 MBq [^18^F]ciprofloxacin, **(B)** 55.1 MBq [^11^C]mephobarbital, **(C)** 6.9 MBq [^18^F]FDG, **(D)** 57.9 MBq (*R*)-[^11^C]verapamil, **(E)** 39.7 MBq [^11^C]tariquidar and **(F)** 97.4 MBq [^11^C]MC113. In contrast to (B), (C), (D), (E) and (F), the variable integration time was not active during the TAC record in figure (A) causing much larger variation of the values. All TACs are corrected for radioactive decay.

The manual sampled and flow-through detector measured blood TACs appeared to be nearly identical as shown in Figure 9 for six different tracers. The advantage of the detector over the manual sampling is best demonstrated by Figure 9A. Here, the count rate measured in the small blood samples by the gamma-counter was very low as compared to the blank measurement, leading to high fluctuations in the blood TAC. Moreover, in Figure 9A, an un-smoothed curve is illustrated, where the variable integration time was not active during the TAC record causing much larger variation of the values.

**Figure 10:**
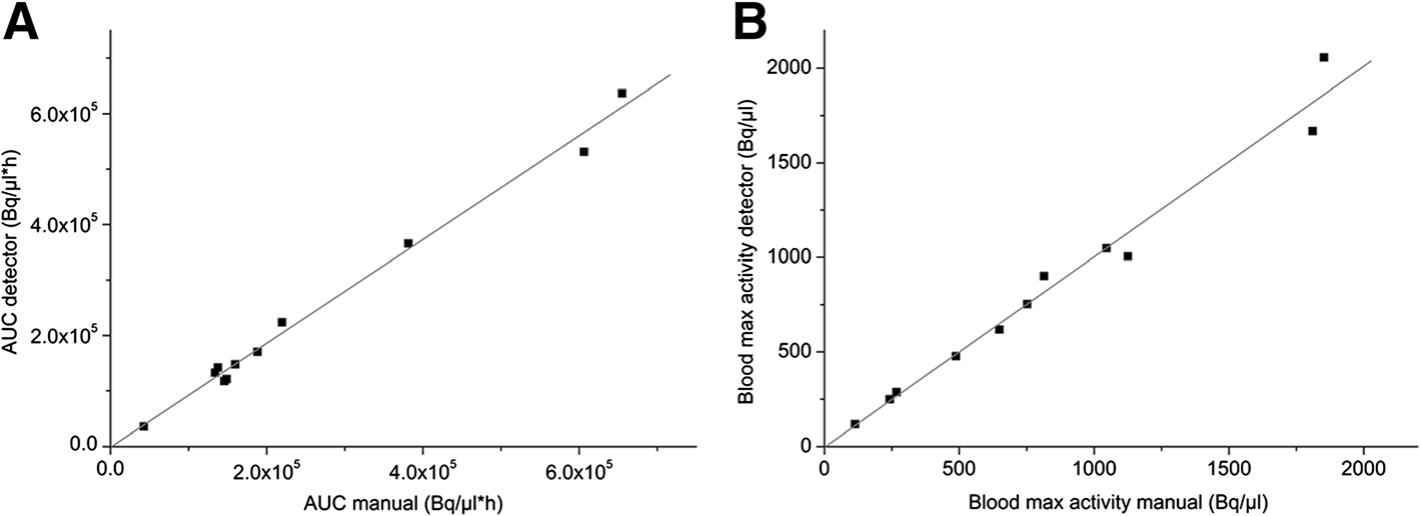
**The correlation of the AUC and peak TAC values obtained by manual sampling and from the flow-through detector.** Correlation between the **(A)** calculated AUCs derived from the manual and detector measured input function and **(B)** maximum blood radioactivity measured manually or by the flow-through detector. Each data point represents one studied animal. The grey line corresponds to the linear fit.

The correlation of the AUC and peak TAC values obtained by manual sampling and from the flow-through detector is shown in Figure 10. The regression lines calculated for the 11 measured animals were *y* = −1378.53 + 0.94*× (*r* = 0.996; *p* < 0.0001) and *y* = −4.20 + 1.01*× (*r* = 0.988; *p* < 0.0001), respectively. The mean differences in the AUC and peak TAC values between the manual and detector-based input functions were 9.3% ± 9.7% (max 24.1%, min −3.2%) and −0.4% ± 7.0% (max 12.0%, min −9.9%), respectively.

Animals did not exhibit any obvious adverse effects caused by the arterio-venous shunt for an observation period of up to 90 min. In the terminal blood samples, we sometimes observed a slight pink colouring of the plasma pointing to haemolysis which we relate to the physical force caused by the peristaltic pump.

## Discussion

We successfully developed a high-sensitivity flow-through detector working in coincidence mode with extremely low background sensitivity. The developed device can be operated via an arterio-venous shunt and was evaluated with six different radiotracers in rats. By using the arterio-venous shunt, the blood loss can be reduced to a minimum, which is in contrast to most proposed blood flow-through detection systems for small animals that operate only in one way [19,23,33,34]. Similar shunt systems which work by coincidence detection [18,35,36] have been proposed before. In comparison to those systems, we have demonstrated that our system can be operated not only with [^18^F]FDG but with a variety of ^18^F- and ^11^C-labelled molecules, especially also with lipophilic radiotracers such as (*R*)-[^11^C]verapamil and [^11^C]tariquidar.

The Teflon tubing led to good reproducible results and avoided adsorption of the lipophilic tracers to the tubing wall. Before finally using Teflon®PFA, we have tested Tygon R3607 and Tygon silicone Pt3350, where we observed severe adhesion to the inner walls of the tubes especially with the lipophilic CNS tracers. Even pre-treatment of the whole tubing system with a high concentration of unlabelled compound did not reduce this effect.

The whole tubing system, starting from the venous and arterial catheters, to the Teflon tubing and the Pharmed®BPT tube inside the peristaltic pump have an inner diameter of 0.51 mm to minimize turbulences and unsteady blood flow.

With a flow rate of 840 μl/min and a blood volume inside the sensitive detection volume of 33.4 μl, it takes about 2.4 s for the blood to pass the detector. This time is comparable with the time it takes to collect a manual sample in a 20 μl microtube. The detection volume of the presented system is larger as the detection volume from another dedicated animal blood sampling systems (twilite system from Swisstrace GmbH), where a volume inside the counter of only 6 μl was reported [36].

Dispersion in the measured input function can produce large errors in quantitative PET. Reported external dispersion caused by the arterial detector system in a clinical study was around 10 s and led to a 33% error in the measured cerebral blood flow [37]. Votaw and Shulman [16] reported on a dispersion constant of 1.3 s in their work leading to a quantitative error of only 0.3%. As the dispersion values obtained in our measurements are around 1.6 to 2 s, we are slightly worse as compared to the later study. Still, as we do not observe a distinct difference in the shape between the manually sampled and automatically measured blood input curve, it can be expected that dispersion does not play a dominant role in our measurements.

The performance of the detector exhibits an excellent agreement with the manually measured blood input curve - ranging from an injected activity of 5.5 to 100 MBq. The sensitivity of 6.5% measured with our system is comparable to the sensitivity of other flow-through detectors such as the Pico-Count (Bioscan, Inc., Washington, DC, USA) detector which has a reported sensitivity of 6.9% [16] or the microvolumetric β blood counter [38] with calculated optimum absolute sensitivities of 7.1% (^18^F) or 21% (^11^C) for the PE50 measurements with comparable inner diameter (0.58 mm).

The measured blood TACs are of high statistical quality, even when injecting only 5.5 MBq and the curves are much smoother as compared to the manual sampling arterial input functions, so that the initial injection peak is not missed. This is of special importance when evaluating radiotracers with fast kinetics and short physical half-life.

Due to the coincidence measurement, we have measured excellent low background count rate of only 1.1 cps for a 185-MBq perturbation source at 10 cm distance and 0.2 cps at 30 cm distance. Another advantage is that the developed system is independent on the nuclide used and can be used for any positron emitting tracer as shown in the present study.

With the developed software, the blood TACs can be smoothed over a defined period of time. Depending on the variability of the count rate, the software chooses the appropriate period of time for integration within a range of 4 to 20 s. Although the smoothing process reduced some temporal information of the curve, we did not observe a serious problem as the smoothing process does not start as long as the count rate increases or decreases constantly.

Our results suggest, that measuring the input function by using the developed flow-through detector consisting of a peristaltic pump, LYSO-PMT detector in coincidence mode and continuous blood sampling via an arterio-venous shunt during the scanning is feasible for the assessment of input functions for a variety of tracers without affecting the physiological status of the animal as a result of abundant blood sampling. The connection of the arterial and venous lines with the detector tubing can be easily disconnected for collecting larger blood samples used for radiometabolite analysis and blood-to-plasma ratio determination. In the given study, a mean total blood volume of 1.8 ± 0.2 ml was extracted from each animal by manual sampling. Without this validation procedure, the extracted blood is only given by the samples for metabolite analysis.

Animals did tolerate the surgical procedure and the arterio-venous shunt well for an observation time of up to 90 min.

## Conclusions

This study demonstrates that the developed blood sampling system can be used for *in vivo* small animal PET studies in rats in a reliable way. The usage of the systems enhances the accuracy of the input curve as handling of small blood samples especially with low activity (as for ^11^C) is prone to measurement errors. Additionally, the radiation exposure of the experimenters can be reduced, as it is not required anymore to continuously draw samples where the personal is in close contact to the radioactive animal and blood.
